# Effect of Bay Leaves Essential Oil Concentration on the Properties of Biodegradable Carboxymethyl Cellulose-Based Edible Films

**DOI:** 10.3390/ma12152356

**Published:** 2019-07-24

**Authors:** Esther Rincón, Luis Serrano, Alina M. Balu, José J. Aguilar, Rafael Luque, Araceli García

**Affiliations:** 1 Departamento de Química Orgánica, Universidad de Córdoba, Campus de Rabanales, Edificio Marie Curie (C-3), CTRA. Nnal. IV-A, Km 396, E-14014 Córdoba, Spain; 2 Departamento de Química Inorgánica e Ingeniería Química, Universidad de Córdoba, Campus de Rabanales, Edificio Marie Curie (C-3), CTRA. Nnal. IV-A, Km 396, E-14014 Córdoba, Spain; 3 Departamento de Microbiología, Universidad de Córdoba, Campus de Rabanales, Edificio Severo Ochoa (C-6), CTRA. Nnal. IV-A, Km 396, E-14014 Córdoba, Spain; 4 Scientific Center for Molecular Design and Synthesis of Innovative Compounds for the Medical Industry, People’s Friendship University of Russia (RUDN University), 6 Miklukho-Maklaya str., 117198 Moscow, Russia

**Keywords:** films, food preservation, carboxymethyl cellulose, bay leaves, essential oil

## Abstract

Films containing bay leaves essential oils (BEOs) were prepared and evaluated for edible packaging applications. The BEOs were extracted by the Soxhlet method, using ethanol or methanol as organic solvent. Then, films were prepared by “solvent casting” technique using carboxymethyl cellulose (CMC), with different concentrations for the as-obtained BEOs (from 1% to 30% wt.). The resulting films were characterized to evaluate their physical (thickness, moisture content, water solubility and water vapor permeability), optical (transparency and UV-light barrier), mechanical (tensile strength and elongation at break), antioxidant and antimicrobiological properties Attractive films were obtained for food active packaging applications, as they presented a high antioxidant activity (up to 99%) and total phenolic content, and good barrier properties against water vapor (50% improved of CMC) in the case of CMC-film containing 15% wt. ethanolic extract. Related to optical properties, UV-light barrier effect was increased (almost 100% of protection) avoiding typical lipids oxidation in food systems. High water solubility (93%) was also found, ensuring also their biodegradability. Moreover, it was demonstrated that developed films inhibit microorganisms’ growth (*Escherichia coli* and *Candida glabrata*), this avoiding an early food oxidation.

## 1. Introduction

Food can be protected against mechanical damage, chemical agents and microbiological activity through packaging materials. Therefore, packaging is the main key to preservation, distribution and commercialization of food products [1]. Synthetic plastics based on petrochemicals (low cost, good mechanical and barrier properties) are commonly used as food packaging materials [2] but they have produced serious environmental problems and the price of oil is getting higher. For this reason, in recent years, biodegradable packaging materials have been the focus of much research [3,4].

Synthetic polymers could be replaced by bio-based biodegradable polymers since they are renewable [5]. Biopolymers have been used to prepare packaging films [6,7]. Cellulose is a renewable source and its derivatives have excellent properties to form films. Water-soluble cellulose derivatives such as carboxymethyl cellulose (CMC) can form a continuous matrix. For this reason, CMC is extensively used in pharmaceutical and food industry. This polymer is also edible allowing its use as a packaging product [8].

An edible film (EF) is a thin layer of edible material, which, once formed, can be placed on or between food components. [9]. EFs are efficient ways to protect fresh food during storage. They can be used to manage permeability to water, oxygen, carbon dioxide or to inhibit the degradation produced by lipids or micro-organism growth in a food system [10,11].

Some properties, such as water adsorption or UV-light barrier, are important when developing packaging films. Packaging films must keep humidity levels in a packaging product [12]. Moreover, water adsorption capacity indicates the swelling capacity of the film in contact with food, liquids, or during storage. For this reason, water adsorption can affect the mechanical properties [13]. On the other hand, UV-light is responsible of the lipids oxidation which produces undesirable flavours and odours. Therefore, a film with UV-light scavenging components can contribute to delay this oxidation process [14]. 

Besides all this, films containing antimicrobial agents can affect the growth of pathogenic microorganisms [15,16,17]. Several antimicrobial compounds, such as these derived from spices, have been proposed to use in packaging films [18,19]. Spices are rich in flavonoids and phenolic acids [20]. These compounds have antioxidant and antimicrobial properties. In this sense, some authors have described the use of essential oils (EOs) from spices in packaging materials to control microbial contamination. They observed a reduction in growth of *Pseudomonas spp.* and *Escherichia coli O157:H7* in meat [21].

About 3000 plant EOs are known, but just 300 are commercially important in the market of flavours and fragrances [22]. Bay leaves EO (0.8% to 3%) contains mainly 1,8-cineol (up to 50%), in addition to eugenol, acetyl, methyl-eugenol, α and β-pinene, phellandrene, linalool, geraniol and terpineol [23]. Additionally, antioxidant and antimicrobial effects of this oil are known. The addition of bay leaves EO to edible films could contribute to the improvement of the properties of these films. 

The aim of this study is focused on the development of edible films with CMC and different concentrations of bay leaves essential oil. The obtained films were characterized to determine their chemical structure, physical, mechanical and optical properties. Moreover, the antioxidant capacity and total phenolic content was measured, and the films’ antimicrobial activity was tested for their suitability for food packaging.

## 2. Materials and Methods

### 2.1. Materials

Bay leaves samples were obtained from laurel tree pruning wastes, kindly supplied by an independent farmer from Córdoba (Spain). CMC (average molecular weight of 90,000) was purchased from Sigma-Aldrich. All other reagents used in this work were of analytical grade.

### 2.2. Bay Leaves Essential Oil Extraction

Bay leaves essential oils (BEOs) were obtained by Soxhlet extraction using ethanol (E-BEOs) or methanol (M-BEOs) as solvents. Prior to the extractions, bay leaves were manually washed and dried at 55 °C. Then, they were ground in a coffee grinder and stored in a dry place until use.

Ground bay leaves (5 g) were extracted with 200 mL of the solvent of choice (ethanol or methanol) for 5 h. After cooling to room temperature, extracts were collected and vacuum concentrated to minimize the amount of solvent. 

### 2.3. Bay Leaves Essential Oil Characterization

To determine the composition of these BEOs (directly related to their potential use in food packaging), the as-obtained extracts were analysed by gas chromatography (GC) in an Agilent Technologies 7890 A GC System (Agilent, Madrid, Spain) chromatograph equipped with a Petrocol^TM^ DH (100 m × 0.25 mm × 0.50 μm) column (Agilent, Madrid, Spain) and a flame ionization detector (Agilent, Madrid, Spain) (FID). Three calibration curves were performed to quantify the three main compounds present in the extracts (eucalyptol, linalool, eugenol) [24]. Moreover, total phenolic content (TPC) in the extracts was determined according to the Folin-Ciocalteu method [25]. Antioxidant activity of BEO samples and reference compounds (eucalyptol, linalool and eugenol) was measured according to the ABTS (2,2’-azino-bis(3-ethylbenzthiazoline-6-sulfonic acid) method [26]. Folin-Ciocalteu method and ABTS assay were conducted in triplicate.

### 2.4. Preparation of Films

Films were prepared as described by Dashipour et al. [27] with some modifications. CMC film (used as control film) was prepared by dissolving 0.5 g of CMC in 100 mL (0.5% w/v) of distilled water under continuous magnetic stirring at 60 °C for 40 min. The forming dispersion was cast in the centre of levelled glass plates. Then, it was dried at 60 °C for 30 h. Films containing BEOs were obtained by adding E-BEO or M-BEO to the CMC solution to achieve final concentrations of 1%, 5%, 10%, 15%, 20% and 30% (w/w%). Before characterization, films were conditioned at 25 °C and 50% relative humidity (RH) for three days. Resulting films were labelled according to the extract concentration and the BEO used in their preparation as: 1E-BEO, 5E-BEO, 10E-BEO, 15E-BEO, 20E-BEO, 30E-BEO, 1M-BEO, 5M-BEO, 10M-BEO, 15M-BEO, 20M-BEO and 30M-BEO.

### 2.5. Fourier Transform Infrared Spectroscopy (FTIR)

Obtained films were characterised by attenuated total reflectance infrared spectroscopy (ATR-IR) according to the methodology described by García et al. [26]. The test was carried out by direct absorbance in a single-reflection ATR System (ATR top plate fixed to an optical beam condensing unit with ZnSe lens) with an MKII Golden Gate SPECAC instrument (Kent, UK). Spectra were recorded over 20 scans with a resolution of 4 cm^−1^ in a wavenumber range between 4000 and 400 cm^−1^.

### 2.6. Physical Properties of Prepared films 

Film thickness was determined according to the methodology described by Rojas-Graü et al. [28] with a micrometre Digital Micrometer IP65 0-1”, Digimatic, Mitutoyo (Neuss, Germany) with a sensitivity of 0.001 mm. Results were expressed as an average of at least three random locations for each film.

Moisture content was expressed as the difference in weight of films before and after drying. A laboratory oven was employed at 110 °C until constant weight was reached (dry sample weight). Two replications of each film were used to calculate the moisture content.

Solubility in water (WS) is defined as the percentage of the dry film that solubilizes in water after 24 h [29]. In this study, WS was determined according to the method of Dashipour et al., [27]. The initial film dry weight (*W_0_*) was achieved by drying at 110 °C to constant weight. Then, film samples (a square of 16 cm^2^) were immersed into 50 mL of distilled water for 6 h at 25 °C. After filtration, an undissolved film was placed back into the oven to reach a constant weight to obtain the final dry weight of the film (*W_f_*). Three replications of each films were used and WS was calculated according to the following Equation (1):

(1) $$\mathrm{WS}\left(\%\right)=\frac{W_{0}-W_{f}}{W_{0}}\;\times 100$$

Water vapour permeability (WVP) of the films was determined according to ASTM E96/E96M-10 standard [30]. From each film a square of 4 cm^2^ was taken for the test. This measurement was conducted in triplicate.

Plastic containers were used, whose lids were perforated with a circle of 100 mm in diameter, later covered with film squares, adhered with aluminium adhesive tape. A desiccant material (CaCl_2_) was added to the vessel. Then, containers were placed in an enabled chamber that remained at 25 °C and 50% RH, and weight measurements of the containers were taken at the beginning and for 24 h noting the mass gain.

With these data and through the formulas obtained from the ASTM E96 standard, the passage of water vapour (and, therefore, permeability) of the films was quantified. The Equations (2) and (3) used were:

(2) $$WVTR=\frac{G}{t\times A}$$

where *G* is weight gain (g) during a time *t* (h) through a material with an area *A* (m^2^), being *WVTR* water vapour transmission rate and,

(3) $$WVP=\frac{WVTR\times thickness}{P\times H}$$

where, *thickness* is the film thickness analysed (m), *P*, saturated vapour pressure (Pa), *H*, humidity in the room (expressed as a per one), being *WVP* the water vapour permeability coefficient. 

### 2.7. Mechanical Properties 

To measure the mechanical properties of film samples, a testometric tensile tester, model LF Plus Lloyd Instrument (AMETEK Measurement & Calibration Technologies Division, Largo, FL, USA), was employed. These properties included Young Modulus (YM), load at break (LB) and elongation at break (ELB) according to the ASTM standard method D882 [31]. Results were expressed as the average of four samples. Before measurements, all the film strips (1.5 cm × 10 cm) were equilibrated at 25 °C and 50% HR. Then they were fixed between the grips with an initial separation of 65 mm, and the crosshead speed was set at 10 mm/min and 1 kN load cell.

### 2.8. Optical Properties 

The ultraviolet and visible light barrier properties of the films were analysed in a spectrophotometer Perkin Elmer UV/VIS spectrometer Lambda 25 (Waltham, Massachusetts) using transmittance mode in UV-VIS regions (200–800 nm). This analysis was conducted in duplicate. Transparency and UV-barrier property were determined according to the Equations (4) and (5) proposed by Han [32]:

(4) $$Transparency=\frac{log\%T_{660}}{x}$$

where *%T_660_* is the percent transmittance at 660 nm and *x* is the film thickness (mm).

(5) $$UV-barrier=100-\left(\frac{\%T_{280}}{\%T_{660}}\right)\times 100$$

where *%T280* is the transmittance at 280 nm and *%T660* is the transmittance at 660 nm.

### 2.9. Antioxidant Activity (ABTS assay)

Antioxidant activity of the films was evaluated according to the same method in Section 2.3. [26] but using 1 cm^2^ of the film to reduce the ABTS radical. Briefly the radical solution (7 mM ABTS in 2.45 mM potassium persulphate) was prepared and left to stand in the dark at room temperature for 14 h before using. This solution was then diluted with ethanol to an absorbance of 0.70 ± 0.02 at 734 nm. The analysis of the samples was carried out by mixing 4 mL of the radical solution with the film, reading the absorbance at 734 nm against ethanol. Absorbance of all samples was recorded after 6 min and the reduction of ABTS, or antioxidant power AOP (%), was determined following the Equation (6) proposed by García et al., with some modifications [26]:

(6) $$AOP=\%reduction\;of\;ABTS_{734\;nm}=\frac{A_{734,ABTS6’}-A_{734,film6’}}{A_{734,ABTS0’}}\;\times 100$$

where *A*_734*,ABTS6’*_ is the absorbance at 734 nm of the radical solution after 6 min, *A*_734*,film6’*_ is the absorbance at 734 nm of the sample after 6 min and *A*_734*,ABTS0’*_ is the initial absorbance (0 min) at 734 nm of the radical solution before the 6 min.

The measurement of the antioxidant activity of the film samples was carried out in triplicate.

### 2.10. Total Phenolic Content (TPC)

The total phenolic content (TPC) of prepared films was estimated according to the method proposed by Dashipour et al. [27]. This analysis was conducted in triplicate. First, a calibration curve with gallic acid (reference compound) was constructed (0–25 mg/L) to express the TPC results as mg gallic acid equivalents (GAE/g) per gram of dried film. For prepared film, 25 mg of each were dissolved into 5 mL of distilled water and then 0.1 mL of this solution was mixed with 7 mL of distilled water, 0.5 mL of *Folin-Ciocalteu* reagent, 1.5 mL of sodium carbonate (saturated solution) and distilled water until a final volume of 10 mL. The mixture was kept for 30 min in a thermostatic bath at 40 °C. Finally, the absorbance of the samples was read at 765 nm against water on a UV spectrometer. This procedure was conducted in triplicate. *%TPC* was calculated following the next Equation (7):

(7) $$\%\;TPC=\frac{A/C}{1000\;\times \;\left(\frac{5}{M}\right)}\;\times 100$$

where *%TPC* is the percentage of total content of phenolic compound per grams of dried film, *A* is the absorbance of the sample at 765 nm, *C* is the concentration of gallic acid (GA) obtained from the standard calibration curve (mg GA/L), and *M* is the weight of dried film (g).

### 2.11. Antimicrobial Activity of Prepared Films

Antimicrobial activity of film samples was determined following the agar diffusion method with two replications for each film sample*. Escherichia coli* and *Candida glabrata* strains (from an own collection) were used as model microorganisms that typically attack food. Film samples were placed in the centre of the plate previously inoculated with the microorganisms. The cultures were grown for 48 h at 28 °C for *C. glabrata*, and 37 °C for *Escherichia coli*, on nutrient agar and YPD (Yeast Extract-Peptone-Dextrose) media, respectively. 

### 2.12. Statistics

Physico-chemical analyses of the films were carried out at least in duplicate, reporting the results as mean values ± s.d. The influence of the extraction solvent and BEO concentration in the film formulations on their properties was assessed by performing several one-way analyses of variance (ANOVA) using the software STATGRAPHICS centurion XV (Madrid, Spain), version 15.2.11 (StatPoint, Inc.) where differences at P < 0.05 were considered significant. The Fisher’s least significant difference (LSD) method was used to discriminate among means for Multiple Range Tests. Moreover, a Multiple-Variable Analysis was carried out in order to evaluate possible interactions or/and correlations between all the analysed parameters (except for the type of solvent used for BEO obtaining). A more detailed description of the procedure is given as Supplementary Materials.

## 3. Results and Discussion

### 3.1. BEOs Characterization

As result of the extraction process, different yields were obtained using ethanol or methanol (32.61% and 16.67% extraction yield, respectively). The final concentration of the obtained extracts was 150 g/L for E-BEO and 65.3 g/L for M-BEO.

According to data reported by other authors [24], eucalyptol, linalool and eugenol are three of the main compounds present in bay leaves extracts. In this study, BEOs were obtained by Soxhlet extraction using ethanol or methanol as solvents. These extracts were analysed by GC and it was found that E-BEO presented high contents of linalool, eugenol and eucalyptol but together with other active compounds (elemicin, methyleugenol, caryophyllene), whereas M-BEO presented the highest content of these three main compounds. Thus, the extraction with methanol resulted the one that gave the greatest extraction yield of these compounds (Table 1).

Related to the antioxidant power (AOP), E-BEO and M-BEO showed results greater than 60% (Table 2). But it was the E-BEO that exhibited the highest antioxidant capacity. Moreover, if TPC is analysed, it can be seen that M-BEO resulted in a low content comparing to the E-BEO (48.34 and 83.41 GAE/g BEO, respectively). This was due to the fact that although methanol extracted higher amount of the three compounds quantified in this study, ethanol is extracting other antioxidant phenolic compounds such as elemicin or methyleugenol, which are contributing to the antioxidant activity and the total phenolic content. In methanolic extracts, these other active compounds were found but with a relative abundance less than 1%, so their contribution to the M-BEO properties was considered not significant. Moreover, according to the AOP obtained for the three main compounds present in the extracts, it is just eugenol the one that is contributing to the AOP. This is because of the phenolic structure of this compound.

These results suggested that E-BEO should further improve some films properties (antioxidant and antimicrobial) as it has higher AOP and TPC.

### 3.2. Chemical Structure

**Figure 1:**
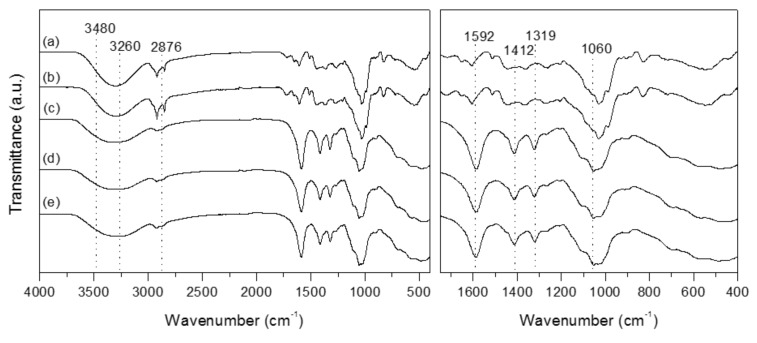
Full (left) and magnified (right) Attenuated Total Reflectance Infrared Spectroscopy (ATR-IR )spectra of BEO samples and prepared films with different BEO concentrations. (**a**) E-BEO; (**b**) M-BEO; (**c**) carboxymethyl cellulose (CMC) film (control); (**d**) 15E-BEO film; (**e**) 30M-BEO film.

Molecular interactions were investigated using ATR-IR technique (Figure 1). The stretching frequency of the COO group is demonstrated by the absorption band at 3260 cm^−1^ [33] with overlaps with the −OH stretching region at 3480–3440 cm^−1^. Bands at 2876 cm^−1^, 1412 cm^−1^ and 1319 cm^−1^ are due to the C–H stretching, −CH_2_ scissoring and −OH vending vibrations, respectively. The band at 1060 cm^−1^ is assigned to CH–O–CH_2_ stretching [34]. Peaks around 1592 cm^−1^ are attributable to antisymmetric vibration of COO− groups [35,36].

It seems both the control film (CMC) and, for example, the 15E-BEO film or 30M-BEO film present a very similar spectrum, independent of the presence and concentration of the extract in the film. Figure 1a,b show spectra of E-BEO and M-BEO. As can be seen, the most representative peaks of these spectra coincide with those of the control film. For this reason, no differences are observed between the films, since the peaks and bands of the essential oil are masked with those of the carboxymethyl cellulose. Very similar results were obtained by Wen et al. [37], where the characteristic adsorption peaks of the cinnamon essential oil were not observed in FTIR spectrum of polyvinyl alcohol/cinnamon essential oil/β-cyclodextrin antimicrobial nanofibrous film. This indicating that the essential oil was efficiently included in the matrix. 

### 3.3. Physical Properties

**Figure 2:**
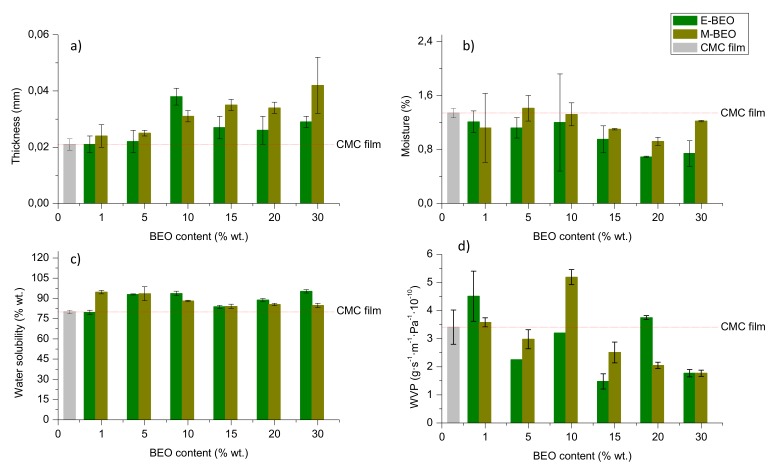
Physical properties of CMC films formulated with different concentrations of BEO: **a**) thickness (mm); **b**) moisture content (% wt.); **c**) solubility in water (% wt.) and **d**) water vapor permeability (WVP, g·s^−1^·m^−1^·Pa^−1^·10^−10^).

Physical properties (thickness, moisture content, WS and WVP) of control film and BEO-incorporated films are shown in Figure 2. Film thickness (Figure 2a) ranged from 0.021 to 0.042 mm, showing a clear linear relationship with the concentration of BEO (See Tables S1 and S2 of Supplementary Materials). The entrapment of BEO microdroplets into the CMC matrix could be the explanation for this behaviour. Dashipour et al., [27] reported this same behaviour for CMC films incorporating *Zataria multiflora* EO.

Previous studies about moisture content in films [38,39,40] have shown that, normally, the incorporation of EOs in polymeric matrices entails the increase of the hydrophobicity in films. The interaction of the EO components and hydroxyl groups of the CMC could reduce their availability to interact with water molecules so a decrease in film moisture content with the oil concentration occurs. In the present work (Figure 2b), it has been found that moisture content in films containing BEO varies from 0.70% to 1.22% independently of the BEO concentration. However, as the amount of EO in the film increases, their solubility in water increased (93%) ensuring their proper biodegradability (Figure 2c). The type of extract used in film formulation (E-BEO or M-BEO) resulted to have no influence on moisture behavior (Table S3) as well as variations of 1% to 10% wt. did not significantly affect this property (Table S1).

One of the most important factors to make proper food packaging is the WVP property. Depending on the food to be protected, the film should be capable to inhibit or to reduce the moisture transfer from the atmosphere. As shown in Figure 2d, the control film exhibited 3.41 ± 0.61 (g·s^−1^·m^−1^·Pa^−1^·10^−10^) of WVP. The presence of BEO, either when ethanol or methanol were used during the extraction, led to better barrier properties of the prepared films. The WVP for the 15E-BEO film was 1.48 ± 0.27 (g·s^−1^·m^−1^·Pa^−1^·10^−10^), reducing the water vapour transmission more than 50% of the control film. For those films containing M-BEOs, this occurred at 30% wt. (1.77 ± 0.11 g·s^−1^·m^−1^·Pa^−1^·10^−10^). In general, the presence of BEO increased the hydrophobicity ratio in the films, reducing their water vapour permeability and then enhancing their barrier properties. WVP values in the case of 10-BEO and 20E-BEO films can be explained since according to Carsi et al., [41] and Raskoya et al., [42], there are several factors than can affect the essential oil effect in a polymeric matrix such as the glass transition temperature of the polymeric matrix, the content of essential oil and the biocomposites processing technique. Moreover, changes in the molecular structure can significantly modify the physical properties of a polymeric material. The confinement and the existing interactions between the polymer and essential oils highly affect the dynamics and statics of biocomposites polymers. Essential oils can cause “attractive or repellent” interactions with the polymeric matrix creating peaks in the properties with the added concentration (Figure S1). 

### 3.4. Mechanical Properties

**Figure 3:**
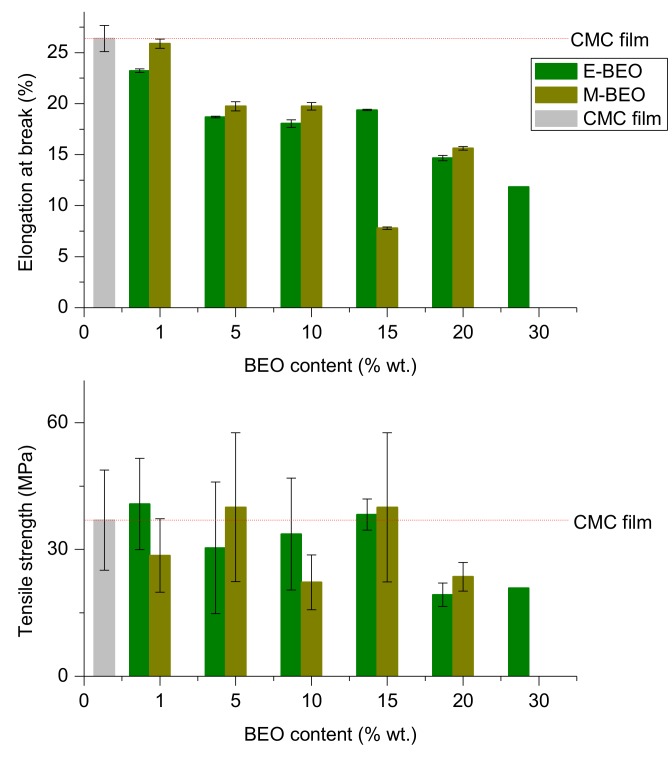
Mechanical properties of CMC films formulated with different concentrations of BEO: Bottom-tensile strength (MPa), top-elongation at break (%).

When considering the application of a food packaging material, TS (elongation resistance, MPa) and ELB (stretching capacity, mm) must be taken into account [38]. Figure 3 shows the effect of BEO incorporation on the mechanical properties of CMC films. TS and ELB ranged from 19,30 MPa to 40.76 MPa and 7.79% to 26.38%, respectively. Tensile strength appeared to be not affected by the nature of the extract (Table S3) but by the amount of extract incorporated to the film. BEO addition in large quantities (20%–30% wt.) involved the obtention of very fragile films. This meant a markedly reduction of TS, out of the equipment measurement range. Again, the 15E-BEO film showed the best result since it obtained a value of 38.26 MPa compared to 36.96 MPa in the control film.

Same result was obtained for 15M-BEO film. A cross-linking effect is produced by a strong interaction between the polymer and the low concentration of BEO. Other researchers have reported this behaviour for chitosan-EO and chitosan-oleic acid films [43,44]. In contrast, at the highest concentration of EO, TS is decreased; this difference can be attributed to weaker carbohydrate-oil interaction in higher concentrations [45].

The effect of type of extract used or its concentration in the film had a strong effect on its elongation behaviour (Table S1 and S2). The presence of extract resulted on that the film was stiffer the higher BEO concentration. 

### 3.5. Optical Properties

**Figure 4:**
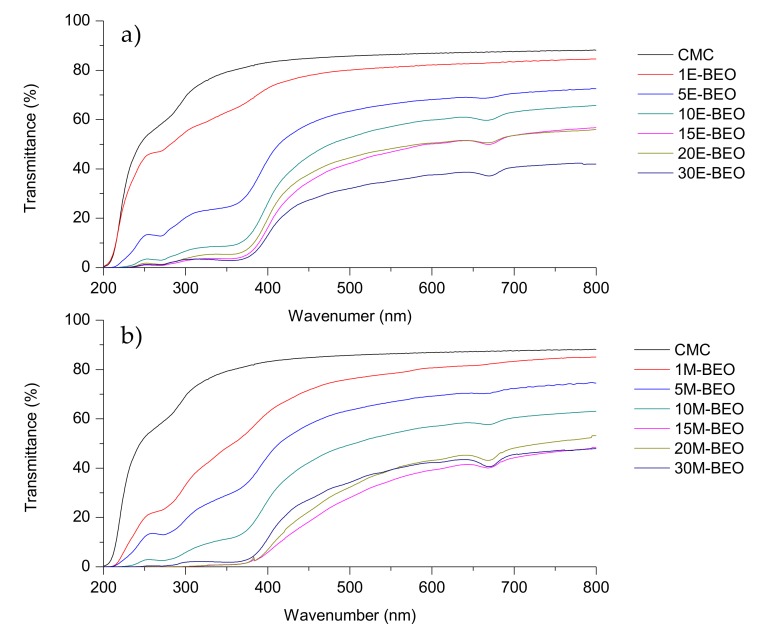
UV-VIS Spectra profile of CMC films formulated with different concentrations of BEO extracted with (**a**) ethanol and (**b**) methanol.

One of the common degradation initiators in food systems is the UV light (200–280 nm) because it produces oxidation of lipids. Therefore, it is important to pay attention to this phenomenon in the food active packaging field [14]. The transmittance of light in the range of UV and visible light for the evaluated films is shown in Figure 4.

**Figure 5:**
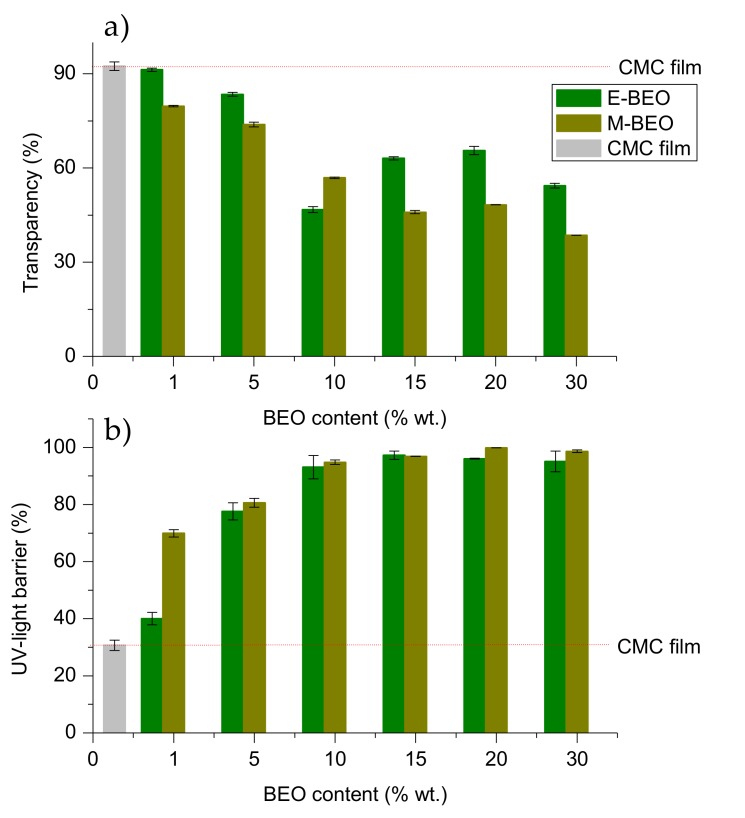
Optical properties of CMC films formulated with different concentrations of BEO: (**a**)transparency (%); (**b**) UV-light barrier transparency (%).

As it can be seen, the protective effect against UV light increased with the amount of BEO in the films, agreeing with previous studies in dialdehyde-CMC crosslinked gelatin edible films [46]. This effect resulted more pronounced in films with E-BEO than in those with M-BEO as demonstrated by the performance of transparency and UV-light barrier calculations (Figure 5). This behavior is probably due to the phenolic compounds present in E-BEO and M-BEO. This type of compounds absorb light at low wavelengths. In fact, these results agree with the data obtained in the characterization of the extracts. As previously mentioned, E-BEOs resulted in higher TPC than M-BEO because ethanol extracts, in addition to eugenol, other phenolic compounds. This same result has been reported by other authors in CMC films also incorporating natural vegetal essential oils [27].

**Figure 6:**
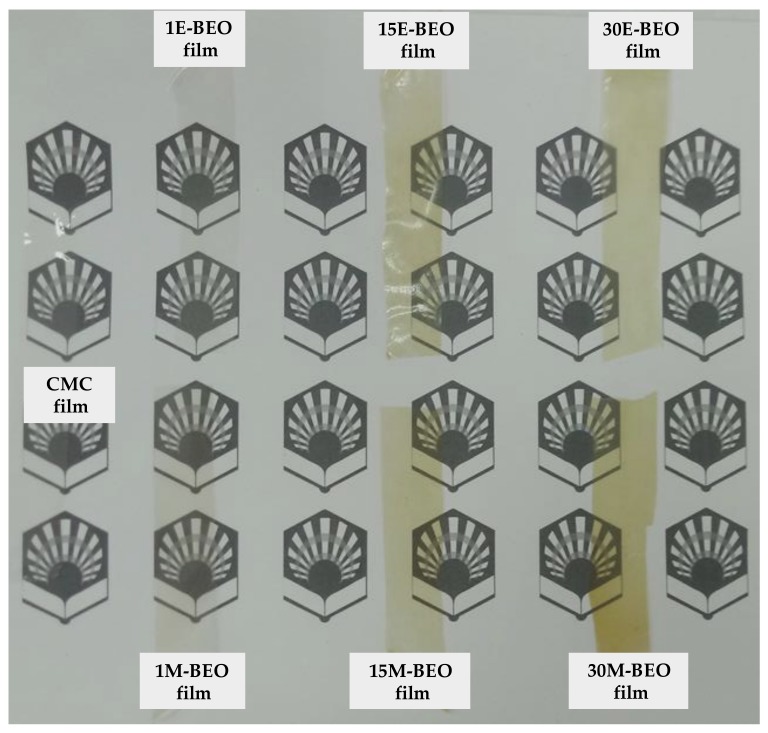
CMC films formulated with different concentrations of BEOs.

When the BEO is present, transparency is decreasing with respect to the control film (92.31 ± 1.48%), acquiring a greenish hue (Figure 6) and increasing the UV-light barrier effect with a maximum of 97.23 ± 1.45% for 15E-BEO and 99.86 ± 0.03% for 20M-BEO. The greenish hue is due to the chlorophyll a of the EO and it can be associated with the increase in peak at 670 nm. At this wavenumber it absorbs the colour red and reflects the green one [47]. A remarkable decrease in transparency was also observed by Teixeira et al. [48] incorporating clove, garlic and origanum EOs in fish protein films. 

### 3.6. Antioxidant Capacity and Total Phenolic Content

**Figure 7:**
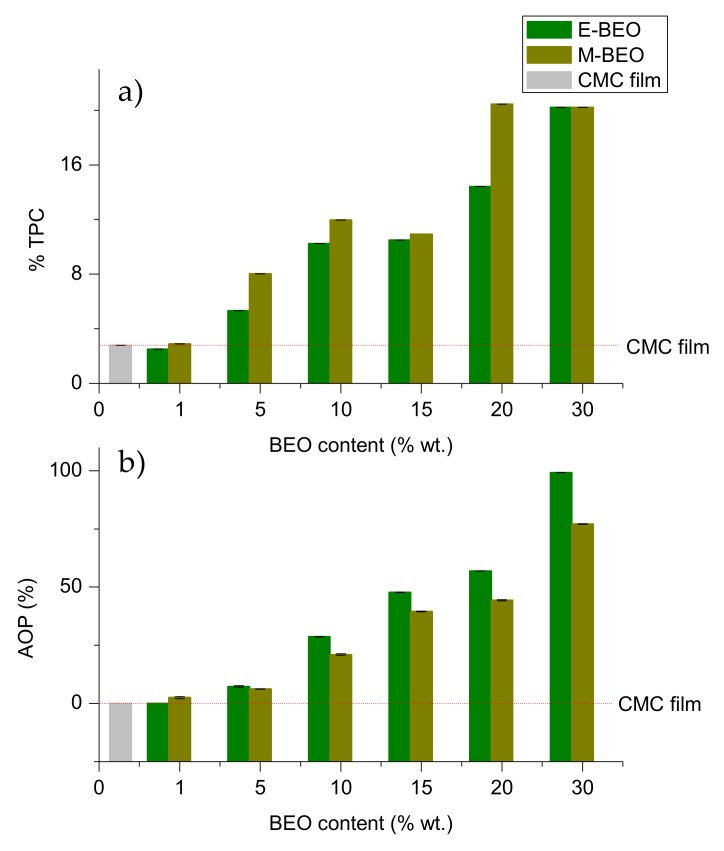
(**a**) Total phenolic content (TPC) and (**b**) antioxidant (AOP) of CMC films formulated with different concentrations of BEO.

The antioxidant activity of EOs has been previously reported [35,49], as well as their use in edible films, that suggested that the antioxidant power (AOP) of these biodegradable films is related to their EO concentration [40,50,51]. Figure 7 shows the AOP and the TPC of different prepared films. Control film (CMC film) and films containing 1% of BEO (1E-BEO and 1M-BEO) resulted in the lowest TPC (<2.90% wt.), as expected. TPC content increases with an increase in BEO concentration. The highest TPC (20.45% wt.) was observed in 20M-BEO.

The control film and 1E-BEO film did not show AOP, and this property markedly increased with an increase in BEO concentrations. Films containing E-BEO at a level of 30% had the highest antioxidant activity (99.23%). Phenolic compounds are responsible for the AOP of EOs in quenching free radicals [25]. These results agree with the BEOs characterization commented in Section 3.1. E-BEO exhibited higher AOP and TPC than M-BEO. Therefore, its incorporation into films should result in greater antioxidant capacity due to phenolic compounds in E-BEO films. 

### 3.7. Antibacterial Activity 

Terpenes and phenolic compounds present in BEO have been reported to be responsible of high antibacterial activity against *Staphylococcus intermedius, Klebsiella pneumoniae* and *Staphylococcus aureus* [52]. Therefore, it would be expected that the inclusion of this essential oil in films would result in inhibition of the growth of certain microorganisms. 

The results obtained in the microbiological assays showed that, in general, films are capable to inhibit, partially or totally, the growth of certain microorganisms. In Figure S2 it is observed as much in the case of *Escherichia coli* as of *Candida glabrata*, the presence of the film produces a strong inhibition in the growth of the colonies with respect to the controls but without being able to observe any growth pattern (inhibition halo). These results agreed with similar studies showing that carvacrol and thymol present in oregano EO incorporated into a calcium caseinate WPI-CMC film significantly inhibited *Escherichia coli* growth [21]. The biocide activity presented by prepared films indicates that the use of BEO should be suitable for the development of active food packaging systems with renewable origin.

### 3.8. Potential Applications

**Figure 8:**
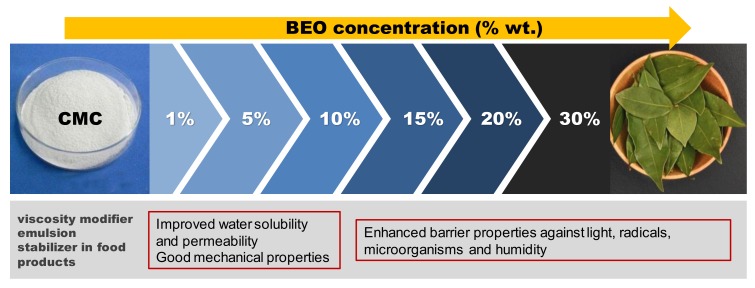
Potential applications of formulated BEO-CMC films.

According to the results obtained from the properties evaluation and the statistical analysis, different applications to the edible films formulated with different amount of bay leaf extract could be proposed (Figure 8). CMC is commonly used as food and cosmetic additive due to its rheological features (emulsifier or viscosity modifier) [53]. In this work, with the addition of BEO during film formulation (even at low % wt.) a clear improvement on water solubility was achieved, leading to an early degradation of the biocomposite formed, which could contribute to reduce the environmental pollution commonly produced by non-biodegradable plastics. 

Moreover, BEO concentration below 10% wt. resulted more rigid films with good water vapour permeability, indicating that these formulations could be suitable for food protecting films. As the amount of extract increased, better barrier properties were obtained against light, microorganisms, radicals, etc. Thus, these formulations could be proposed for the development of special food or cosmetics encapsulation, for which an exhaustive control of environmental factors (light degradation, fungal attack, toxic molecules scavenging) is required.

## 4. Conclusions

In the present work, carboxymethyl cellulose films incorporated with bay leaves essential oil (from 1% to 30% wt.) have been prepared by solvent casting technique.

Results have shown that as the amount of BEO increases, some properties such as thickness and solubility on water (up to 93% wt.) also become higher, ensuring the reduction of environmental pollution. It was shown that the 15E-BEO and 30M-BEO films halved the water vapour permeability property compared to the CMC film. This fact indicated the clear effect of extracting solvent on the final properties of films containing vegetal extracts. M-BEO presented a higher variety of compounds, but E-BEO presented compounds with better antioxidant and antimicrobial properties. Considering this, ethanol can be proposed as the best extraction solvent for bay leaves essential oil obtention. 

Tensile strength and elongation at break of CMC film resulted slightly improved when E-BEO was incorporated at concentrations below than 10% wt. Moreover, the amount of BEO resulted directly related with optical barrier properties of the films, increasing the UV-light scavenging up to 97% in the case of 15 E-BEO film. This should contribute avoiding typical oxidation mechanisms of lipids, thus increasing its shelf-life of food. The TPC in films significantly increased with increasing BEO concentration, as well as the antioxidant activity. Finally, it has been demonstrated that prepared films are able to partially or totally inhibit the growth of certain microorganisms that typically attacks food (*Escherichia coli* and *Candida glabrata*). For all these reasons, 15E-BEO film could be the best option for food packaging application if the addition of a plasticizer is considered in order to improve the mechanical properties obtained at this concentration. In future works, the optimization of the BEO extraction process for films as food packaging additive will be studied. 

## Figures and Tables

**Table 1:** Eucalyptol, linalool and eugenol amount in bay leaves essential oil extracted with ethanol (E-BEO) and methanol (M-BEO) by gas chromatography (GC) analysis.

-	Eucalyptol (mg)	Linalool (mg)	Eugenol (mg)
E-BEO	6.0	4.0	3.0
M-BEO	12.0	7.0	6.0

**Table 2:** Antioxidant power (AOP) and total phenolic content (TPC).

Sample	AOP (%)	TPC (GAE/g BEO)
E-BEO	74.73 ± 4.71	83.41 ± 4.19
M-BEO	62.13 ± 0.71	48.34 ± 3.51
Eugenol	91.5 ± 0.53	n.m.
Linalool	n.d.	n.m.
Eucalyptol	n.d.	n.m.

## Supplementary Materials

The following are available online at https://www.mdpi.com/1996-1944/12/15/2356/s1, Figure S1: Scatterplot matrix displaying interactions between different dependent variables (properties) of the analysed films and the factor BEO CONCENTRATION (% wt. in the film), Figure S2: *Escherichia coli* growth in (a) control culture media and (b) in the presence of 15E-BEO film. *Candida glabrata* growth in (c) control culture media and (d) in the presence of 15M-BEO film, Table S1: ANOVA table and MRT results for each variable evaluated at seven levels of the factor BEO CONCENTRATION, Table S2: Correlation found between each pair of analysed variables, Table S3: ANOVA table and MRT results for each variable evaluated at three levels of the factor SOLVENT.
