# Supplementary material for: Effect of Bay Leaves Essential Oil Concentration on the Properties of Biodegradable Carboxymethyl Cellulose-Based Edible Films
Source: Materials (Basel). 2019 Jul 24;12(15):2356. doi: 10.3390/ma12152356 (PMC6695984; doi:10.3390/ma12152356)
Supplement: Supplementary file 1 [file materials-12-02356-s001.pdf]

materials

Effect of bay leaves essential oil concentration on the properties of biodegradable CMC-based edible films

SUPPLEMENTARY DATA

Statistical evaluation of results

S.1. One-way ANOVA analysis

The statistical one-way ANOVA analysis, using the software STATGRAPHICS Centurion XV, version 15.2.11 (StatPoint, Inc.), was conducted for all the properties (dependent variables) analysed for the formulated films. This study was performed considering the variance of these variables respect to two fixed factors, i.e., the type of solvent used during the bay leaves extraction process and the concentration of extract incorporated to each film.

For both set of analyses, in ANOVA study the F-test evaluated whether there are significant differences amongst the means of the variables. The more the means of the dependent variable differ between the groups of the independent variable, the higher the value of F, indicating that there are more differences and therefore a stronger relationship among the variables. Moreover, when $P < 0.05$, differences were considered significant at the 95.0% confidence level.

To determine which means are significantly different from which others, a Multiple Range Tests were also carried out. As result, homogenous groups of data considering different levels can be identified, and thus stablishing how each factor (level) affect to each variable. In this work, the Fisher's least significant difference (LSD) method was used to discriminate among means for Multiple Range Tests (MRT).

S.1.1. ANOVA analyses considering the type of solvent

Two different solvents were used for the extraction of bay leaves: ethanol and methanol. Considering that blank film samples contained only CMC (without extract), this means that ANOVA of each variable was conducted for 3 levels of the factor SOLVENT (no extract, ethanol extracted, and methanol extracted). **Table S1** shows the results of ANOVA and MRT for each analysed variable (39 pairs of comparisons) with the type of solvent.

S.1.2. ANOVA analyses considering the extract concentration in the film

Each CMC film formulation was prepared at 7 different concentrations of extract (0, 1, 5, 10, 15, 20 and 30% wt. in the film). Thus, ANOVA of each variable was conducted for 7 levels of the factor BEO CONCENTRATION. **Table S2** shows the results of ANOVA and MRT for each analysed variable (39 pairs of comparisons) with the amount of extract incorporated in the film.

S.2. Multiple Variable Analysis

A Multiple-Variable Analysis was carried out using the software STATGRAPHICS Centurion XV, version 15.2.11 (StatPoint, Inc.) in order to evaluate possible interactions or/and correlations between all the analysed parameters (except for the type of solvent used during BEO obtaining). The results are displayed in **Figure S1** and detailed in **Table S3**, where Pearson product moment correlations between each pair of analysed variables are shown. For computing each coefficient 39 pairs of data values were used. These correlation coefficients range between -1 and +1 and represent the strength of the linear relationship between the variables (inverse and direct relationships, respectively). The number in parentheses corresponds to the P-value which tests the statistical

significance of the estimated correlations. P-values below 0.05 indicate statistically significant non-zero correlations at the 95.0% confidence level.

Table S1. ANOVA table and MRT results for each variable evaluated at 7 levels of the factor BEO CONCENTRATION.

VARIABLE	F-RATIO	P-VALUE	LEVELS OF THE FACTOR BEO CONCENTRATION % wt. (*)						
			0	1	5	10	15	20	30
**Thickness**	6.00	0.0003							
**Moisture**	2.40	0.0500							
**Water solubility**	4.32	0.0027							
**WVP**	9.79	0.0000							
**Tensile strength**	4.70	0.0016							
**Elongation at break**	18.20	0.0000							
**Transparency**	29.91	0.0000							
**UV light barrier**	62.88	0.0000							

(*) Within each row (variable), levels containing the same colour form a group of means within which there are no statistically significant differences, i.e., the behaviour of variables results statistically homogeneous within this group of means.

Table S2. Correlations found between each pair of analysed variables.

	TENS. STREN.	ELONG. AT BREAK	TRANSP.	UV LIGHT PROTECT.	BEO CONCENT.	THICKNESS	MOISTURE	WATER SOLUB.	WVP
TENS.		0.5252 (0.0006)	0.4285 (0.0065)	-0.3312 (0.0394)	-0.5648 (0.0002)	-0.2345 (0.1507)	0.4394 (0.0051)	0.0247 (0.8813)	0.2547 (0.1177)
ELONG. AT	0.5252 (0.0006)		0.7954 (0)	-0.3312 (0.0394)	-0.8454 (0)	-0.6812 (0)	0.2093 (0.2011)	0.0212 (0.8981)	0.5085 (0.0009)
TRANSP.	0.4285 (0.0065)	0.7954 (0		-0.8563 (0)	-0.7875 (0)	-0.8334 (0)	0.2000 (0.2221)	-0.1306 (0.4281)	0.3914 (0.0138)
UV LIGHT	-0.3312 (0.0394)	-0.3312 (0.0394)	-0.8563 (0)		0.7330 (0)	0.6408 (0)	-0.2687 (0.0982)	0.3901 (0.0141)	-0.3992 (0.0118)
BEO	-0.5648 (0.0002)	-0.8454 (0)	-0.7875 (0)	0.7330 (0)		0.5931 (0.0001)	-0.4199 (0.0078)	0.0892 (0.5894)	-0.5461 (0.0003)

THICKNESS	-0.2345<br>(0.1507)	-0.6812<br>(0)	-0.8334<br>(0)	0.6408<br>(0)	0.5931<br>(0.0001)		0.1438<br>(0.3824)	0.1160<br>(0.4818)	-0.2232<br>(0.1719)
MOISTURE	0.4394<br>(0.0051)	0.2093<br>(0.2011)	0.2000<br>(0.2221)	-0.2687<br>(0.0982)	-0.4199<br>(0.0078)	0.1438<br>(0.3824)		0.0042<br>(0.9796)	0.3238<br>(0.0443)
WATER	0.0247<br>(0.8813)	0.0212<br>(0.8981)	-0.1306<br>(0.4281)	0.3901<br>(0.0141)	0.0892<br>(0.5894)	0.1160<br>(0.4818)	0.0042<br>(0.9796)		-0.0552<br>(0.7387)
WVP	0.2547<br>(0.1177)	0.5085<br>(0.0009)	0.3914<br>(0.0138)	-0.3992<br>(0.0118)	-0.5461<br>(0.0003)	-0.2232<br>(0.1719)	0.3238<br>(0.0443)	-0.0552<br>(0.7387)	

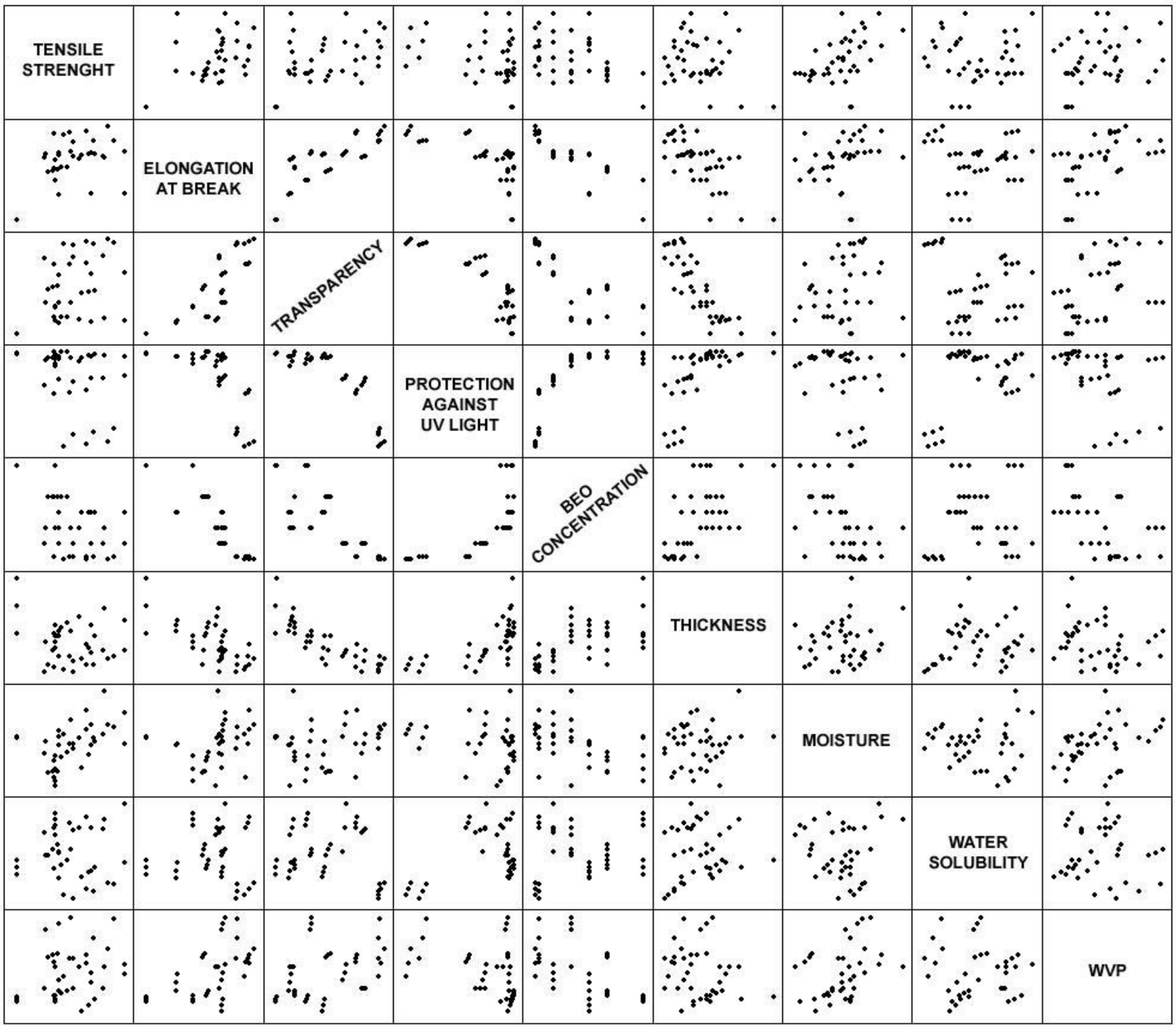

Figure S1. Scatterplot Matrix displaying interactions between different dependent variables (properties) of the analysed films and the factor BEO CONCENTRATION (% wt. in the film).

Table S3. ANOVA table and MRT results for each variable evaluated at 3 levels of the factor SOLVENT.

**VARIABLE**	**F-RATIO**	**P-VALUE**	**LEVELS OF THE FACTOR SOLVENT(*)**		
			No solvent	Ethanol	Methanol
**Thickness**	4.28	0.0215			
**Moisture**	2.99	0.0631			
**Water solubility**	3.88	0.0298			
**WVP**	0.37	0.6949			
**Tensile strength**	1.04	0.3646			
**Elongation at break**	4.14	0.0240			
**Transparency**	7.54	0.0018			
**UV light barrier**	16.61	0.0000			

(*) Within each row (variable), levels containing the same colour form a group of means within which there are no statistically significant differences, i.e., the behaviour of variables results statistically homogeneous within this group of means.

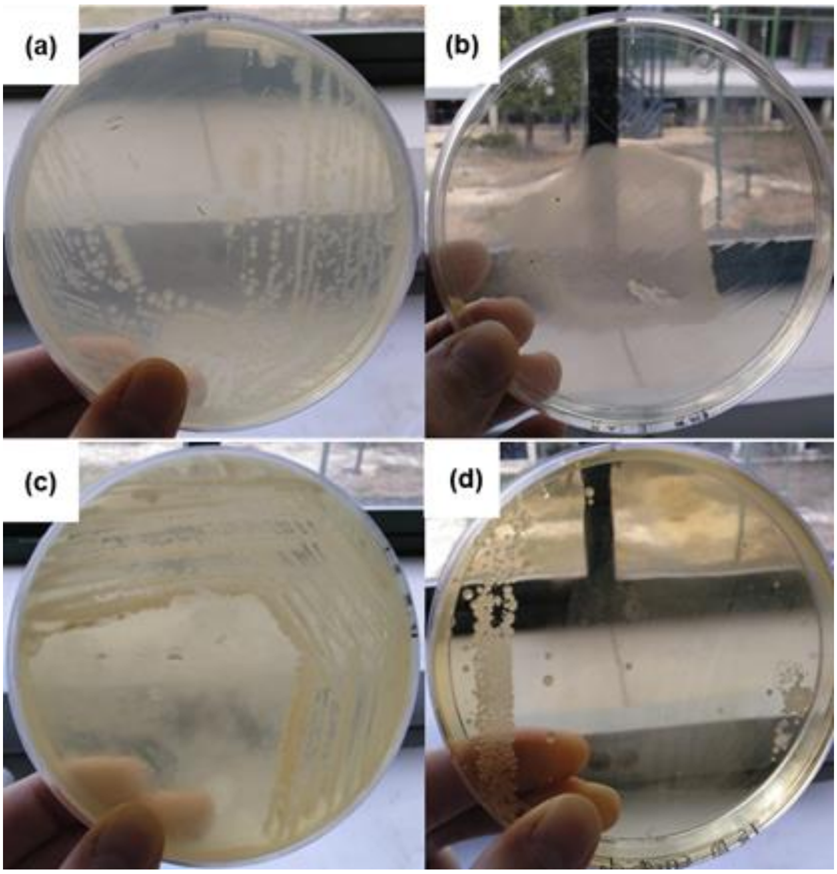

Figure S2. *Escherichia coli* growth in (**a**) control culture media and (**b**) in the presence of 15E-BEO film. *Candida glabrata* growth in (**c**) control culture media and (**d**) in the presence of 15M-BEO film.
